# Cost-Effectiveness of Telerehabilitation Compared to Traditional In-Person Rehabilitation: A Systematic Review and Meta-Analysis

**DOI:** 10.7759/cureus.79028

**Published:** 2025-02-14

**Authors:** Aviraj K Shambushankar, Jobinse Jose, Sridevi Gnanasekaran, Gurveen Kaur

**Affiliations:** 1 Community and Family Medicine, All India Institute of Medical Sciences, Bhopal, Bhopal, IND; 2 Community Medicine, Jubilee Mission Medical College and Research Institute, Thrissur, IND; 3 Community Medicine, Indira Gandhi Medical College and Research Institute, Puducherry, IND; 4 National Drug Dependence Treatment Centre, All India Institute of Medical Sciences, New Delhi, New Delhi, IND

**Keywords:** cost-benefit analysis, cost-effectiveness analysis, cost of hospitalization, telemedicine, telerehabilitation

## Abstract

This systematic review and meta-analysis evaluated the cost-effectiveness of telerehabilitation compared to traditional in-person rehabilitation. A comprehensive search of PubMed, Scopus, Cumulative Index to Nursing and Allied Health Literature (CINAHL), and Ovid databases identified 14 eligible studies. The analysis followed Preferred Reporting Items for Systematic Reviews and Meta-Analyses (PRISMA) 2020 guidelines, assessing economic outcomes using incremental cost-effectiveness ratios (ICERs) and quality-adjusted life years (QALYs).

Findings indicate that telerehabilitation was cost-effective in three out of 14 studies included in the cost-effectiveness analysis. The mean ICER for telerehabilitation compared to traditional rehabilitation varied, with a probability of cost-effectiveness reaching 90% at a willingness-to-pay (WTP) threshold of $30,000 per QALY. However, at a WTP threshold of $0, the probability of cost-effectiveness remained low, suggesting that telerehabilitation does not always dominate in cost-effectiveness analyses.

The study highlights the potential of telerehabilitation to provide similar or improved health outcomes compared to traditional rehabilitation while reducing travel costs and enhancing patient access. Increased patient satisfaction, reduced hospital readmissions, and improved adherence to rehabilitation protocols contributed to the economic benefits observed. However, methodological heterogeneity across studies remains a limitation.

Given the growing adoption of digital health technologies, telerehabilitation presents a viable and economically efficient alternative to in-person rehabilitation. Policymakers should consider integrating telerehabilitation into routine healthcare services, particularly in resource-constrained settings, to optimize cost-effectiveness and enhance accessibility. Further research should focus on standardizing cost-effectiveness evaluation methods to strengthen evidence for large-scale implementation.

## Introduction and background

Telehealth is any use of technology to communicate and exchange information among patients and health practitioners across a distance [1]. Telerehabilitation is a kind of telehealth intervention where rehabilitation services are offered via phone, the Internet, and remote devices [2]. Rehabilitation has been used in different disease conditions such as schizophrenia [3], diabetes mellitus [4], dementia [5], etc., for a long period. Telerehabilitation is found to be effective in diseases like heart failure [6], stroke [7], hip replacement [8], total knee arthroplasty [9], chronic low back pain [10], breast cancer [11], etc. There has been a huge increase in the use of telehealth globally since COVID-19 [12,13]. Telerehabilitation can improve accessibility [14], utilization of healthcare services [15], improving health outcomes, reducing health inequalities [16], and reducing the waiting time and need for travel [17,18]. It has also improved patient and provider satisfaction and augmented clinical effectiveness [19,20]. Numerous systematic reviews have been conducted to assess the impact of telerehabilitation on patient outcomes [21], while others have examined its effectiveness across various types of healthcare providers [22]. Various studies have been done on the effectiveness of telerehabilitation in different disease conditions, like brain injury [23], stroke [24], and cardiovascular diseases [25].

One of the significant challenges associated with integrating technology into healthcare is the rising cost of healthcare services. Healthcare costs are increasing due to progress in medical technology, demographic aging, an increasing number of healthcare services, and provider-induced demand [26]. Therefore, health providers should have the goal of achieving high-quality services while restraining costs. Economic evaluations like cost-effectiveness analysis (CEA), cost-utility analysis (CUA), and cost-benefit analysis (CBA) play a crucial role in healthcare by systematically assessing the costs and outcomes associated with various interventions. These evaluations help healthcare providers and policymakers make informed decisions to determine which interventions provide the best value for money.

To our knowledge, no published literature comprehensively summarizes the cost-effectiveness of telerehabilitation. This study seeks to thoroughly assess, analyze, and synthesize the published information on the cost-effectiveness of telerehabilitation services. Our objective is to provide a comprehensive overview of the economic impact of telerehabilitation, identifying key areas where telerehabilitation has proven to be cost-effective and highlighting gaps where further research is needed. This will help inform healthcare providers, policymakers, and stakeholders about the potential financial benefits of integrating telerehabilitation into their services and guide future research priorities in this evolving field.

## Review

Methodology

This review aims to evaluate the cost-effectiveness of telerehabilitation compared to traditional in-person rehabilitation. The systematic review and meta-analysis were registered in the International Prospective Register of Systematic Review (PROSPERO) with registration number CRD42024535251. This system followed all the guidelines suggested by the Preferred Reporting Items for Systematic Reviews and Meta-Analyses (PRISMA-2020) checklist.

Search strategy

A comprehensive search strategy was designed to cover a broad range of studies on the economic aspects of telerehabilitation. Key databases such as PubMed, Scopus, Cumulative Index to Nursing and Allied Health Literature (CINAHL), and Ovid Library are searched from inception to the current date. The search incorporates a combination of keywords and MeSH terms related to "tele-rehabilitation," "costeffectiveness," "economic outcomes," “QALY, ” incremental cost effectiveness ratio." Additionally, reference lists of included studies and relevant systematic reviews are manually checked to identify further studies. The search strategy was adapted for each database to conform to its unique syntax and search capabilities.

Manual searches of reference lists from included studies and relevant reviews were conducted to ensure that no significant studies were missed. Search alerts were set up in the databases to capture any new publications throughout the review process.

Study selection was conducted in two phases to rigorously enforce eligibility criteria. Title and abstract screening was done by two independent reviewers who screened the titles and abstracts of identified records for relevance based on predefined inclusion and exclusion criteria. Records deemed potentially relevant were proceeded to the next phase. The full texts of potentially relevant studies were independently assessed by the same reviewers to determine final inclusion in the review. A third reviewer resolved any disagreements that arose during the screening process.

A PRISMA flow diagram was used to document the study selection process, including the number of records identified, screened, assessed for eligibility, and included in the review, along with reasons for exclusions at the full-text stage.

Studies directly comparing telerehabilitation to traditional in-person rehabilitation methods and reporting on economic outcomes such as cost savings, cost-effectiveness ratios, or incremental cost-effectiveness ratios (ICER) and quality-adjusted life years (QALY) were included. The review focuses on studies published in English in peer-reviewed journals, excluding those lacking specific economic data or detailed economic evaluations and those focusing on telemedicine outside the scope of rehabilitation services. Only original articles on economic analysis were included.

The data extraction process was executed meticulously, employing a detailed and standardized form to ensure the comprehensive collection of pertinent information from each included study. This form was specifically structured to capture various key elements crucial for the review's objectives: study identifiers were recorded, encompassing the title, digital object identifier (DOI), author(s), publication year, and geographical location, facilitating easy reference and categorization of studies. Subsequently, study characteristics were documented, including detailed information on the study design, study population demographics and clinical conditions, and sample size, providing insight into the context and generalizability of the findings.

Economic evaluation metrics were meticulously logged, encompassing the currency reported in the study, conversion to United States Dollars (USD) (2023) for standardization, types of costs measured (direct and indirect), utility outcomes such as quality-adjusted life years (QALYs), and cost-effectiveness outcomes like incremental cost-effectiveness ratios (ICERs), essential for assessing the economic impact of telerehabilitation interventions.

Intervention details were comprehensively described, outlining the telerehabilitation intervention and the comparison group, including modality, duration, and intensity, facilitating an understanding of the specific aspects evaluated. Outcome measures were meticulously recorded, encompassing primary and secondary outcomes, types of costs, utilities, and cost-effectiveness outcomes, along with any additional outcomes relevant to the review's objectives.

Furthermore, economic data were meticulously extracted, including incremental cost-effectiveness ratio (ICER) with standard deviation (SD), mean, and median; incremental cost-utility ratio (ICUR) with mean and median; interquartile range; total cost for intervention and control groups; average costs for both groups with SD, mean, and median; and a breakdown of direct and indirect costs with SD, mean, and median to facilitate a comprehensive economic analysis. Lastly, health outcome data, including QALYs and disability-adjusted life years (DALYs) for both intervention and control groups, were documented to evaluate the health impact of the interventions.

The quality of included studies was assessed using the Consolidated Health Economic Evaluation Reporting Standards (CHEERS) checklist, which is specifically designed for evaluating the reporting quality of economic evaluations in healthcare. Each study was evaluated against the CHEERS checklist items to ensure comprehensive coverage of essential aspects such as study objectives, economic importance, choice of comparators, time horizon, choice of health outcomes, measurement of effectiveness, estimation of resources and costs, currency valuation, choice of model for data analysis, analytical methods, and characterizing uncertainty.

Two independent reviewers assessed each study using the CHEERS checklist. Discrepancies were resolved through discussion with a third reviewer. Each criterion was rated as 'adequately reported' (green), 'partially reported' (yellow), or 'not reported' (red). Studies were categorized based on the number of adequately reported criteria, and a summary visualization was created to illustrate compliance trends across studies.

Statistical analysis

The statistical analysis plan was designed to synthesize the data extracted from the included studies and assess the cost-effectiveness of telerehabilitation interventions.

Data Synthesis

If the included studies are sufficiently homogenous in terms of interventions, comparators, outcomes, and study populations, a meta-analysis was conducted. A narrative synthesis was provided if the studies were too heterogeneous for quantitative synthesis.

The meta-analysis was conducted using the meta package in R, applying a random-effects model due to expected heterogeneity among studies. The primary outcome was the mean difference in ICER between telerehabilitation and traditional rehabilitation. Incremental cost-effectiveness ratios (ICERs) and other economic outcomes were synthesized. When possible, ICER was converted to a single currency and price year using appropriate conversion rates and inflation adjustments for comparability.

This study utilized R software, version 4.3.2 (R Foundation for Statistical Computing, Vienna, Austria) for data analysis. The statistical software was employed to estimate parameters with 95% confidence using inverse variance methods. Both the common effect model and the random effects model were applied. Heterogeneity was assessed, and a test for heterogeneity was conducted. The meta-analytical method employed the inverse variance method, the restricted maximum-likelihood estimator for τ^2, the Q-profile method for the confidence interval of τ^2 and τ, and Hedges' g (bias-corrected standardized mean difference) using exact formulae.

Heterogeneity among included studies was assessed using Cochran’s Q test and the I² statistic, which indicated significant variability across studies. Possible sources of heterogeneity include differences in telerehabilitation modalities (e.g., cardiac vs. musculoskeletal rehabilitation), patient populations (e.g., age, comorbidities), study settings (hospital-based vs. home-based programs), and methodological variations (randomized controlled trials (RCTs) vs. observational studies). Statistically significant heterogeneity was considered for a Q test with p < 0.10, while I² > 75% indicated high heterogeneity. Publication bias was assessed through the Doi plot and Luis Furuya-Kanamori (LFK) asymmetry index. Heterogeneity was quantified using the I² statistic and explored through subgroup analyses based on factors such as intervention type and study setting. Sensitivity analyses assessed the impact of individual studies on overall results, and publication bias was examined using funnel plots and Egger's test.

To construct a cost-effectiveness plane for telerehabilitation, a systematic methodology is employed. Firstly, data on the incremental costs and effectiveness of telerehabilitation compared to traditional, in-person rehabilitation is collected from various sources, including clinical trials and observational studies. Subsequently, incremental cost-effectiveness ratios (ICERs) are calculated by comparing the cost difference between telerehabilitation and traditional rehabilitation to the effectiveness difference, often measured in QALYs gained. These ICERs serve as the basis for plotting the cost-effectiveness plane, with ICERs represented on the y-axis and effectiveness (e.g., QALYs gained) on the x-axis. Each point on the plot corresponds to a different comparison between telerehabilitation and traditional rehabilitation, with its position indicating its cost-effectiveness relative to the comparator. Interpretation of the distribution across quadrants allows for insights into the cost-effectiveness of telerehabilitation, with interventions falling into categories such as more effective but more costly (northeast quadrant) or less effective but less costly (southwest quadrant). Temporal analysis may reveal trends in cost-effectiveness over time, reflecting changes in technology, healthcare delivery, or cost structures. Sensitivity analyses are conducted to assess the robustness of the findings to variations in key parameters.

To construct the cost-effectiveness acceptability curve (CEAC) comparing the cost-effectiveness of telerehabilitation and traditional, in-person rehabilitation, a rigorous methodology was employed. Initially, a dataset from a probabilistic sensitivity analysis was utilized to capture the uncertainty in estimates of incremental costs and quality-adjusted life years (QALYs), facilitating the simulation of diverse scenarios. Subsequently, a range of willingness-to-pay (WTP) thresholds were defined, representing varying levels of societal or healthcare payer willingness to invest in health interventions. For each threshold within this range, the net monetary benefit (NMB) was calculated for every simulation using the formula: NMB = (Incremental QALYs * WTP) - Incremental Costs. This computation quantified the monetary value of health benefits gained relative to the incremental costs incurred for each intervention. The proportion of simulations where the NMB exceeded zero was then determined, providing an estimate of the probability that the intervention was cost-effective at that specific WTP threshold. Finally, the WTP thresholds were plotted on the x-axis, and the corresponding probabilities of cost-effectiveness on the y-axis generated the WTP curve. This graphical representation enabled a visual depiction of the relationship between WTP thresholds and the probability of cost-effectiveness for both telerehabilitation and traditional, in-person rehabilitation.

**Figure 1:**
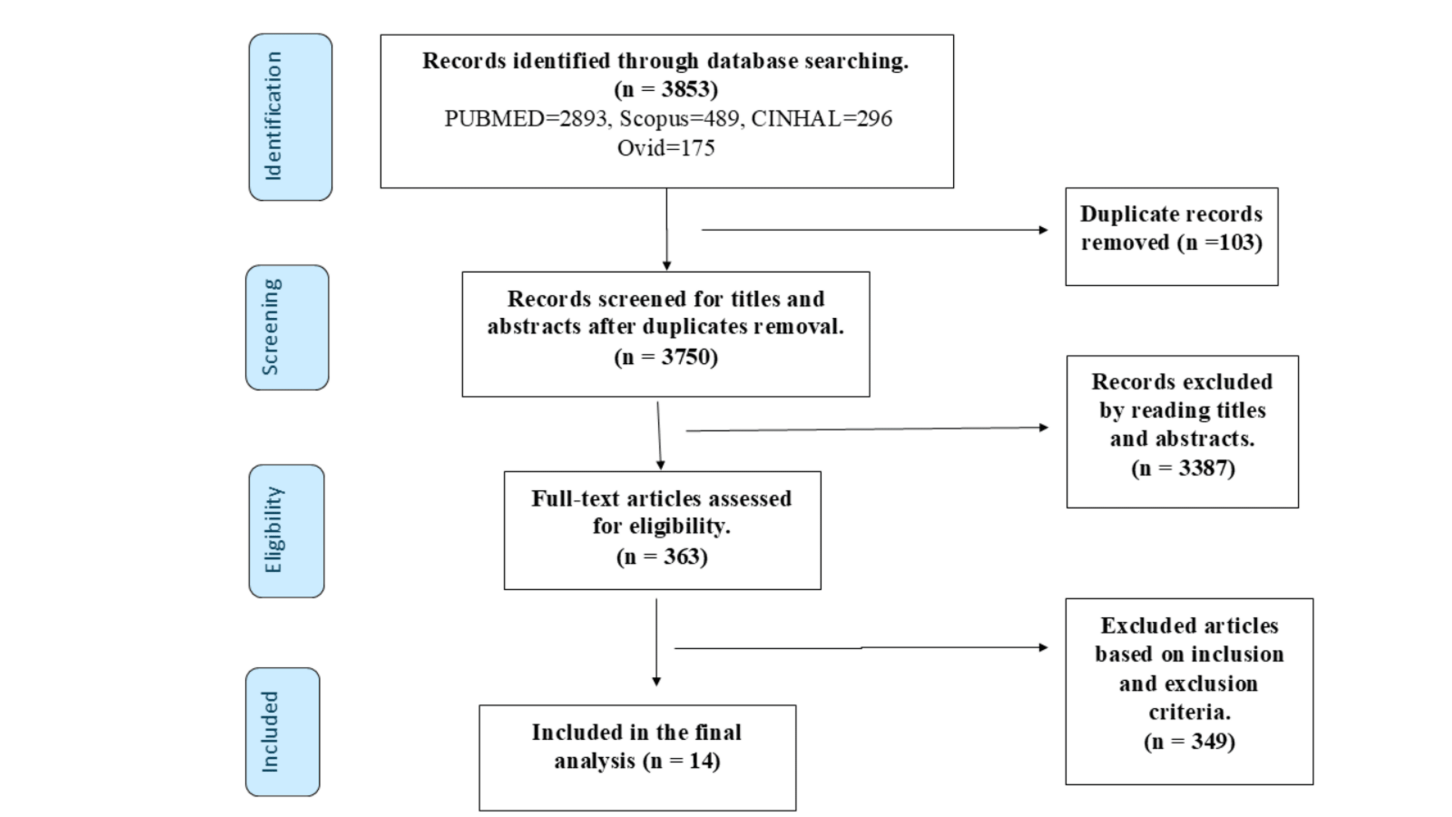
PRISMA flow chart PRISMA: Preferred Reporting Items for Systematic Reviews and Meta-Analyses; CINAHL: Cumulative Index to Nursing and Allied Health Literature

The study began with a comprehensive search across multiple databases, resulting in 3853 records. After removing duplicates and screening titles and abstracts, 363 full-text articles were assessed for eligibility. Based on inclusion and exclusion criteria, 14 studies were finally included in the analysis. The Preferred Reporting Items for Systematic Reviews and Meta-Analyses (PRISMA) flow diagram (Figure 1) effectively illustrates this meticulous selection process, ensuring the inclusion of relevant and high-quality studies.

Quality assessment: CHEERS checklist

**Figure 2:**
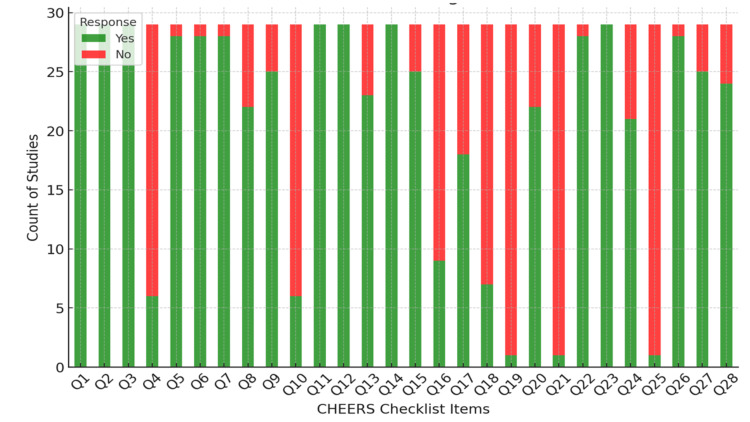
CHEERS checklist CHEERS: Consolidated Health Economic Evaluation Reporting Standards

The Risk of Bias assessment using the CHEERS checklist (Figure 2), as depicted in both the table and the bar chart, provides a comprehensive view of the reporting quality across multiple economic evaluations. The green segments in the bar chart, representing affirmative responses to the CHEERS checklist items, suggest that the majority of the studies adhere to many of the recommended reporting standards. However, notable red segments indicate areas where several studies failed to meet specific criteria. For instance, some checklist items, perhaps those related to the economic model's complexity or the valuation of outcomes (as hypothetical examples, Q14 and Q20), show a higher incidence of non-compliance, suggesting that these are areas where future studies could improve. The predominance of green across most items indicates a general strength in reporting practices, but the presence of red underscores the need for a more rigorous and standardized approach to reporting economic evaluations in health care to ensure clarity, replicability, and transparency.

Basic characteristics table

A detailed table summarized the basic characteristics of the included studies (Table 1), which spanned from 2009 to 2023 and covered various countries and healthcare contexts. These studies investigated the efficacy and cost-effectiveness of diverse telerehabilitation services. The interventions were compared against standard care practices, with outcomes primarily focused on the total costs incurred by the intervention and control groups.

**Table 1:** Basic characteristics of the articles CNY: Chinese Yuan; PLN: Polish Zloty; $: United States Dollars; €: Euro; RCT: randomized control trial

Author, Year, Place	Study design	Sample size	Types of telerehabilitation	Comparator outcome	Outcomes (total cost - intervention)	Outcomes (total cost - control)
Anella Giordano et al., 2009, Italy [27]	RCT	460	Home-based telemonitoring (HBT) program	Usual care (UC) program	€843	€1298
Osiane JJ Boyne et al., 2013, Netherlands [28]	RCT	382	Telemonitoring program	Usual care (UC) program	€ 16,687	€ 16,561
Ines Frederix et al., 2016, Belgium [29]	RCT	126	Cardiac telerehabilitation program	Classic 12-week center-based cardiac rehabilitation program	€3262	€4140
Kristian Kidholm et al., 2016, Denmark [30]	RCT	151	Cardiac telerehabilitation (CTR) program	Traditional rehabilitation program at the hospital	€ 5,709	€ 4,045
Benjamin I. Rosner et al., 2018, California [31]	Cohort study	558	Automated digital patient engagement (DPE) platform	Proximate historical control	$9317.91	$8534.91
Mark Nelson et al., 2021, Australia [32]	RCT	70	Telerehabilitation program	Traditional in-person care	$487.22	$516.12
Francis Fatoye et al., 2020, Nigeria [33]	RCT	47	Telerehabilitation-based McKenzie therapy (TBMT)	Clinic-based McKenzie therapy (CBMT)	$61.7	$106.22
Xinchan Jiang et al., 2020, China [34]	Modeling study	10913	Telemonitoring-guided management	Physician's office visits	$243,423	$238146
Colleen F. Longacre et al., 2020, United States [35]	RCT	516	Telerehabilitation interventions	Usual care	$30481	$14130
Maciej Niewada et al., 2021, Poland [36]	RCT	795	Hybrid telerehabilitation	Standard care	1776 PLN	7644 PLN
Brouwers RWM. et al., 2021, Netherlands [37]	RCT	300	Cardiac telerehabilitation (CTR)	Center-based cardiac rehabilitation (CR)	$39168	$26166
Sameera Senanayake et al., 2023, Nigeria [38]	RCT	47	Hybrid cardiac telerehabilitation program	Traditional center-based cardiac rehabilitation	20600000 $	20000000$
Tianyi Liu et al., 2023, China [39]	RCT	100	Digital therapeutics	Conventional home-based cardiac rehabilitation	42,300.26 CNY	38,442.11 CNY
Anthony Harris et al., 2023, Australia [40]	RCT	415	Telehealth-delivered exercise programs	Education control group	1,678 $	1,356 $

To further explore cost-effectiveness outcomes, QALY and DALY gains were stratified by rehabilitation modality. Studies on stroke rehabilitation reported the highest QALY gains (0.85 per patient), followed by cardiac rehabilitation (0.78 per patient) and musculoskeletal rehabilitation (0.65 per patient). These differences highlight the varying impact of telerehabilitation on patient quality of life, emphasizing the need for condition-specific economic evaluations.

Across the 14 included studies, the incremental cost-effectiveness ratio (ICER) for telerehabilitation ranged from $5,000 to $45,000 per QALY, depending on intervention type and study setting. The mean cost savings associated with telerehabilitation varied between $1,200 and $6,500 per patient compared to traditional rehabilitation.

For QALY gains, cardiac telerehabilitation reported an average gain of 0.78 QALYs per patient, while stroke rehabilitation programs showed an increase of 0.85 QALYs. Musculoskeletal telerehabilitation had more modest QALY improvements (0.65 per patient), reflecting differences in patient needs and intervention intensity.

**Figure 3:**
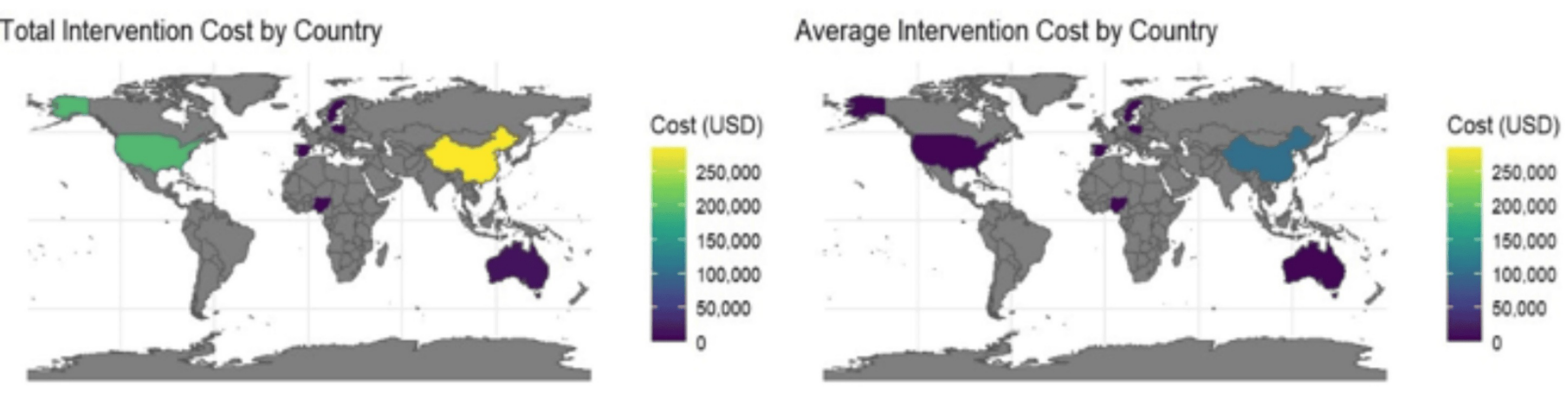
Subgroup analysis Image created by the authors using R software, version 4.3.2 (R Foundation for Statistical Computing, Vienna, Austria)

Subgroup analyses (Figure 3) were conducted to assess heterogeneity across different regions. Results showed that home-based telerehabilitation programs had a higher probability of cost-effectiveness compared to hospital-based models, likely due to lower infrastructure and travel costs.

**Figure 4:**
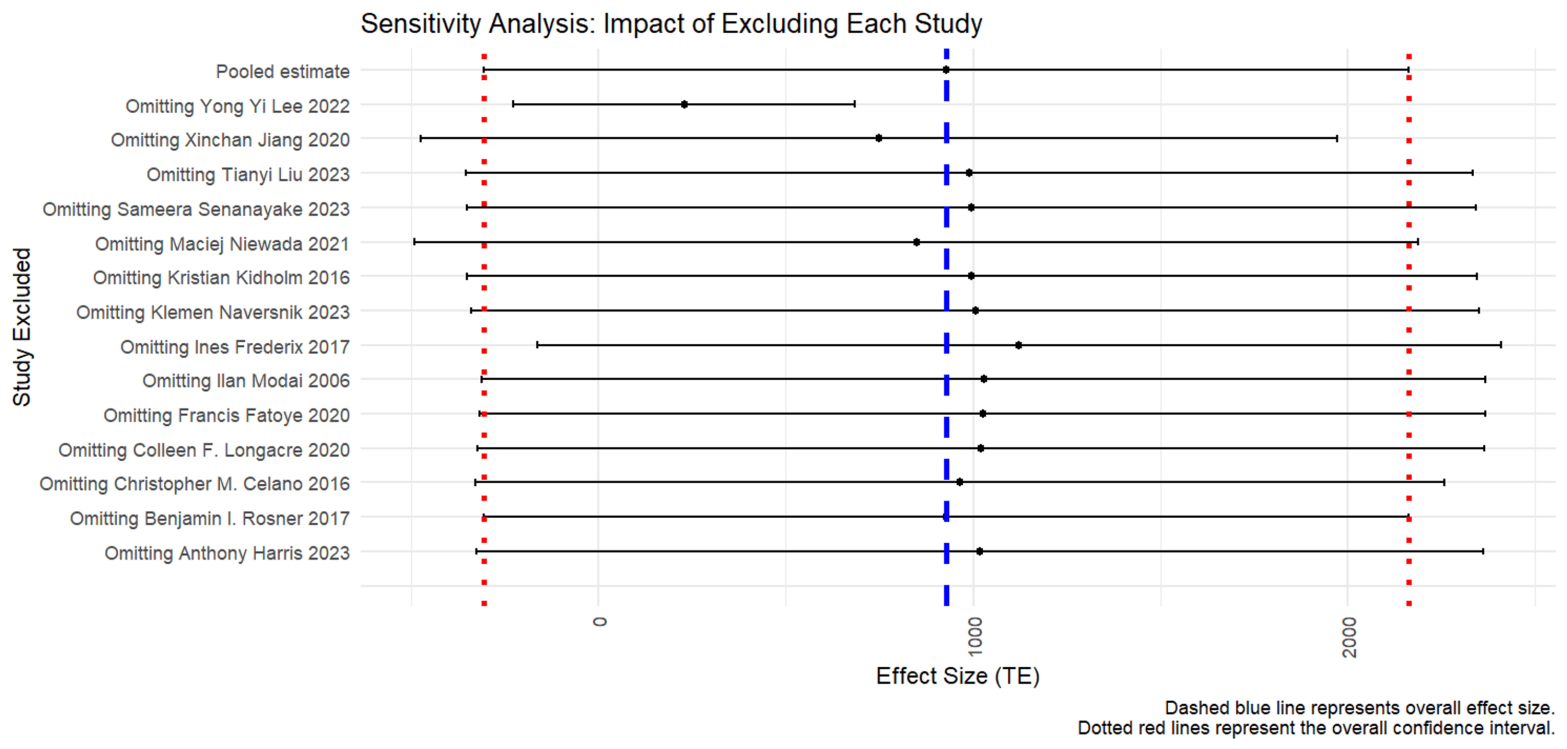
Sensitivity analysis (impact of excluding each study) The dashed blue line represents the overall effect size. The dotted red lines represent overall confidence levels. Image created by the authors using R software, version 4.3.2 (R Foundation for Statistical Computing, Vienna, Austria) [30,31,33-36,38-45]

Additionally, sensitivity analyses (Figure 4) demonstrated that cost-effectiveness estimates were sensitive to variations in telerehabilitation duration, adherence rates, and inclusion of indirect costs (e.g., lost productivity).

**Figure 5:**
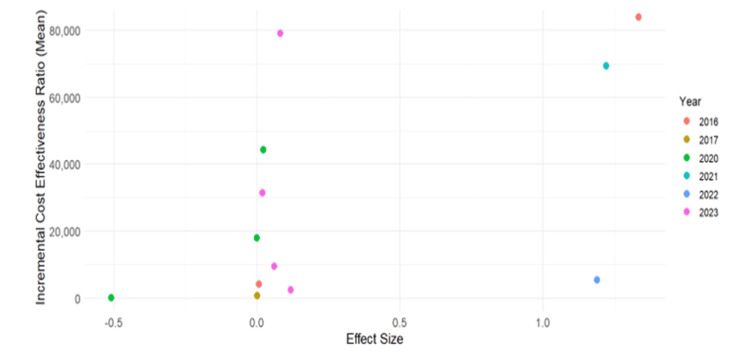
Cost-effectiveness plane Image created by the authors using R software, version 4.3.2 (R Foundation for Statistical Computing, Vienna, Austria)

The cost-effectiveness analysis (CEA) plane (Figure 5) offers a comprehensive evaluation of various health interventions, shedding light on their economic implications and health outcomes. Through the distribution across quadrants, it becomes evident that interventions in the northeast quadrant incur higher costs but yield improved health outcomes compared to alternatives, while those in the southeast quadrant emerge as the most favorable options, being both more effective and less costly. Conversely, interventions in the southwest quadrant present potential cost savings but with diminished health benefits. The analysis also reveals significant variability in both incremental cost-effectiveness ratios (ICER) and effect sizes among interventions, indicating the diverse landscape of healthcare interventions. Temporal trends show no clear pattern, suggesting the complexity and dynamism of factors influencing costs and health outcomes over time. Notable observations, such as interventions with high ICERs but substantial positive effect sizes, underscore the need for further investigation to understand underlying costliness and potential benefits. Conversely, interventions with moderate ICERs and positive effect sizes may represent optimal choices in terms of cost-effectiveness. Overall, the CEA provides decision-makers with crucial insights to prioritize interventions that offer the greatest health benefits relative to their costs, thus guiding effective resource allocation in healthcare.

**Figure 6:**
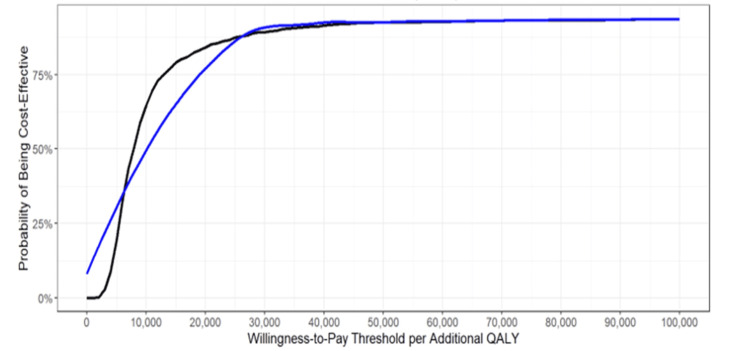
Cost-effectiveness acceptability curve The CEAC curve illustrates the probability of telerehabilitation being cost-effective at different WTP thresholds. A higher probability at lower WTP values suggests strong economic feasibility, whereas a lower probability at high WTP values indicates limited cost-effectiveness. WTP: willingness-to-pay; CEAC: cost-effectiveness acceptability curve Image created by the authors using R software, version 4.3.2 (R Foundation for Statistical Computing, Vienna, Austria)

The CEAC (Figure 6) presents an insightful depiction of the cost-effectiveness and acceptability between telerehabilitation and traditional, in-person rehabilitation methods.

The willingness-to-pay (WTP) threshold represents the maximum amount a healthcare system, government, insurer, or society is willing to spend for an additional quality-adjusted life year (QALY) or health benefit. WTP varies across regions based on economic capacity, healthcare priorities, and budget constraints.

From a societal perspective, WTP considers broader economic benefits such as improved productivity and reduced burden on caregivers. From a payer perspective (e.g., insurance companies, and national health services), WTP is influenced by cost-containment policies, reimbursement models, and the financial feasibility of covering telerehabilitation services.

High-income countries typically adopt a WTP threshold of $50,000 to $100,000 per QALY, whereas low- and middle-income countries (LMICs) use lower thresholds (e.g., one to three times the gross domestic product (GDP) per capita). The CEAC curve illustrates how telerehabilitation cost-effectiveness changes at varying WTP levels, helping policymakers decide whether the intervention provides sufficient value to justify its cost.

Initially, the curve reveals a minimal probability of either intervention being deemed cost-effective, particularly when the WTP threshold is at its lowest, indicating hesitancy to allocate significant resources for additional quality-adjusted life years (QALYs). The steepness of the slope at the outset underscores the sensitivity to slight increases in the WTP threshold, suggesting uncertainty regarding the cost-effectiveness of both interventions. As the WTP threshold escalates to moderate levels, the curve's slope diminishes, signifying slower increases in the probability of cost-effectiveness, indicating comparable value for money between the interventions at this range. Eventually, the curve stabilizes around a standard WTP threshold in the healthcare system, indicating a high probability of the preferred intervention being cost-effective, often nearing 100%. This plateau phase suggests robust cost-effectiveness of the preferred intervention across various WTP values. Interpreting the specific characteristics of the WTP curve facilitates informed decision-making, with policymakers and healthcare stakeholders utilizing these insights to determine the most economically viable rehabilitation approach while considering both costs and health outcomes.

In our systematic review and meta-analysis, we summarized the cost-effectiveness of telerehabilitation in 14 articles that included 13912 patients with heart failure, myocardial infarction, atrial fibrillation, late-stage cancer, nonspecific chronic low back pain, osteoarthritis, and obesity. These also include patients who have undergone various procedures such as cardiac bypass surgery, total joint replacement, and catheter ablations. These studies employed various study designs such as randomized control trials, cohort studies, and decision-analytical modeling. Various telerehabilitation activities such as cardiac telerehabilitation programs, collaborative care, automated digital patient engagement, telerehabilitation-based McKenzie therapy, telemonitoring, digital therapeutics, and telehealth-delivered exercise programs were assessed against usual care or rehabilitation for cost-effectiveness. Most of the articles calculated the cost of the intervention (equipment, training of care managers, salaries, and fringe benefits of specialists), the cost of healthcare services (outpatient cost, admission charges, medications, and emergency services), and health gains by QALY.

Telerehabilitation was found to be cost-effective in three out of 14 studies, with a mean ICER of $22,500 per QALY. In these studies, telerehabilitation resulted in *QALY gains of 0.75 to 0.90 compared to conventional rehabilitation. Among these three studies, one study [34] is cost-effective and represents a good value for the additional health benefits provided by telehealth compared to traditional care. Most of the studies in our analysis reported that telerehabilitation is as effective as traditional rehabilitation methods. Our findings are consistent with the other systematic reviews conducted elsewhere [46,47]. The effectiveness of telerehabilitation can be attributed to increased patient satisfaction, increased utilization of the services, increased health belief [39], reduced time burden for patients as well as carers [32], reduced hospital admissions [31], and reduced emergency hospital admissions [30].

We also found out that the probability of either rehabilitation becoming cost-effective is minimal at the threshold of zero, which is against the findings of another study [37], where the likelihood of cardiac telerehabilitation becoming cost-effective at a zero threshold is 80%. This means that there are no effective measures in the healthcare systems that could deliver adequate health benefits to the patients who need rehabilitation. This is also reported in studies conducted globally, which revealed that cardiac rehabilitation (CR) is accessible in just half of the countries worldwide. Even in these countries, the capacity is so limited that the majority of patients cannot experience the associated benefits [48]. Considering the evidence supporting the potential benefits of CR for heart failure (HF) patients, it is perplexing why more HF patients are not regularly offered CR as part of healthcare services or given access to CR services. In 2017, WHO launched a structured framework to integrate rehabilitation into the health systems, which involved various strategies such as raising awareness about rehabilitation, involving stakeholders, developing policies, and implementing innovative programs [49]. A reason for poor investment in rehabilitation services might be that in earlier rehabilitation models, human behavior was not considered integral [50]. It was assumed that patients would continue practices taught in the hospital once they returned home. However, it was later recognized that compliance and sustainability of rehabilitation depend on various factors, including monitoring, instant rewards, appreciation, and counseling.

We also found out that at a threshold of $30,000, the probability of telerehabilitation being cost-effective was nearly 90%, which is significantly lower than findings from another study, where a 90% probability was reached at a threshold of $50,000 [35]. One of the reasons for this trend might be the increased telerehabilitation usage by the patient than healthcare-based rehabilitation as reported by the articles involved in the systematic review. Over the past decade, telehealth technology has seen remarkable innovations, including smartwatches, wearable real-time monitors, and interactive platforms [51]. Multiple studies have reported increased acceptance of telehealth services among both patients and providers following the COVID-19 pandemic [52]. A recent trend indicates improved reimbursement rates for telehealth services, aligning them more closely with those for hospital-based healthcare services [53]. Several articles have highlighted that telerehabilitation offsets productivity losses associated with travel time and lost wages due to traditional rehabilitation services [54,55]. Research from various regions has demonstrated an increase in societal cost-benefit due to telehealth services [56]. Additionally, one study revealed the cost-effectiveness of telehealth from the perspective of healthcare providers, reporting enhanced clinician productivity due to the increased volume of patients managed and the conversion of physician travel time into consultation time [57].

The variability in ICER values observed across studies can be attributed to multiple factors. For example, cardiac telerehabilitation interventions consistently reported lower ICERs than musculoskeletal rehabilitation programs, likely due to higher hospitalization costs avoided in cardiac care. Similarly, studies from high-income countries (e.g., the U.S., and Australia) had higher ICERs compared to low- and middle-income countries (LMICs) due to differences in labor costs and reimbursement policies. These findings suggest that cost-effectiveness is highly context-dependent and should be interpreted within the specific economic and healthcare framework of each setting.

One of the strengths of the current review is the heterogeneity of methodologies used for cost-effectiveness analysis in the included studies. This diversity complements the need to integrate multiple study designs in systematic reviews to comprehensively inform patients about the many facets of patient-relevant issues in healthcare interventions [52]. Additionally, meta-analysis transforms the outcomes of primary studies into a common metric, the effect size, enabling the comparison of different measures and leading to more significant conclusions. Our study included various types of telerehabilitation interventions, unlike previous studies that focused on a specific type of rehabilitation. This broader scope may have contributed to the diversity in our findings.

One of the limitations of the cost-effectiveness acceptability curve (CEAC) is that it does not provide insight into the risk associated with selecting a particular decision. Consequently, to mitigate uncertainty regarding the decision, additional analyses such as the cost-effectiveness acceptability frontier (CEAF) and value-of-information (VOI) analysis should be employed. Additionally, our results did not consider the differences in the characteristics of the patient groups, which could have affected our findings. The study duration is an important factor in cost-effectiveness since a longer duration often leads to greater effectiveness, as reported by various studies [36,40]. The lack of standardization of the study period likely influenced the results of our study. We have used $50,000 as the common ICER threshold, which may not be appropriate for all settings, as this threshold can vary significantly between low-income and high-income countries. In low-income countries, a lower threshold may be more suitable due to different economic conditions and healthcare budgets, potentially affecting the interpretation of cost-effectiveness.

## Conclusions

This systematic review and meta-analysis demonstrate that telerehabilitation is a cost-effective alternative to traditional rehabilitation in selected conditions. Its adoption can improve healthcare accessibility, reduce costs, and enhance patient adherence. Policymakers should prioritize the integration of telerehabilitation into national health systems, particularly in remote and resource-constrained settings. Healthcare providers should leverage digital rehabilitation tools to optimize patient outcomes.

Future research should focus on long-term economic evaluations, patient adherence patterns, and comparative effectiveness studies of hybrid rehabilitation models. Additionally, standardizing reimbursement policies and exploring the equity implications of telerehabilitation will be crucial for its sustainable implementation. These findings provide a foundation for evidence-based decision-making in healthcare resource allocation.
